# Analysis of normal and dysplastic glenohumeral morphology at magnetic resonance imaging in children with neonatal brachial plexus palsy

**DOI:** 10.1007/s00247-017-3882-1

**Published:** 2017-07-04

**Authors:** Fabian van de Bunt, Michael L. Pearl, Eric K. Lee, Lauren Peng, Paul Didomenico

**Affiliations:** 1 VU University Medical Center, De Boelelaan 1117, 1081 HV Amsterdam, The Netherlands; 2 Kaiser Permanente, 4760 Sunset Blvd Ste. 1213, Los Angeles, CA 90027 USA

**Keywords:** Brachial plexus, Glenoid version, Magnetic resonance imaging, Neonatal brachial plexus palsy, Scapular orientation

## Abstract

**Background:**

Glenoid version and percentage of the humeral head anterior to the scapular line are commonly used 2-D measures to assess deformity of the glenohumeral joint of children with neonatal brachial plexus palsy.

**Objective:**

To assess whether glenoid version and percentage of the humeral head anterior to the scapular line would be altered by standardizing the measurements to the orientation of the scapula.

**Materials and methods:**

Twenty-one bilateral magnetic resonance imaging (MRI) scans were evaluated by four reviewers. Measurements were performed on the axial image slices and again after applying 3-D reformatting.

**Results:**

Three-dimensional reformatting led to intrapatient corrections up to 25° for version and −30% for percentage of the humeral head anterior to the scapular line. The mean difference on the involved side between clinical and anatomical version across all subjects from all reviewers was 2.2° ± 3.9° (range: −4.5° to 11.5°). The mean difference in the percentage of the humeral head anterior to the scapular line after reformatting was −1.8% (range: −15.9% to 5.2%).

**Conclusion:**

Measurements can differ greatly for the same child depending on technical factors of image acquisition and presentation in the clinical setting. With this study, we present a clinically accessible protocol to correct for scapular orientation from MRI data of children with neonatal brachial plexus palsy.

## Introduction

Neonatal brachial plexus palsy frequently results in altered muscular development of the rotator cuff muscles and osseous deformities of the glenohumeral joint [1–7]. Glenoid version and percentage of the humeral head anterior to the scapular line are established 2-D tools to assess deformities occurring to the complex 3-D shape of the scapula and glenohumeral joint [2, 5, 7, 8].

Normal glenoid version in most studies has been reported close to 0°, with values typically ranging less than 10° in either direction, but generally slightly retroverted [9–16]. A large study by Mintzer et al. [17] analyzed version in young children (*n* = 111) and showed similar results finding slightly more retroversion in children between 0 and 2 years old than in children >2 years old, −6.3° ± 6.5° compared to −1.7° ± 6.4°, respectively. In children with neonatal brachial plexus palsy and internal rotation contracture, the glenoid version angle is almost always affected, resulting in increased retroversion [3, 8, 18]. This alters normal glenohumeral mechanics and often leads to subluxation of the humeral head, as measured by percentage of the humeral head anterior to the scapular line. Several classification systems have been developed to grade glenohumeral deformity using these measures, as early osseous changes often lead to severe functional impairment warranting surgical treatment [2, 7]. These classification systems are important for consideration and follow-up of surgical treatment.

**Fig. 1:**
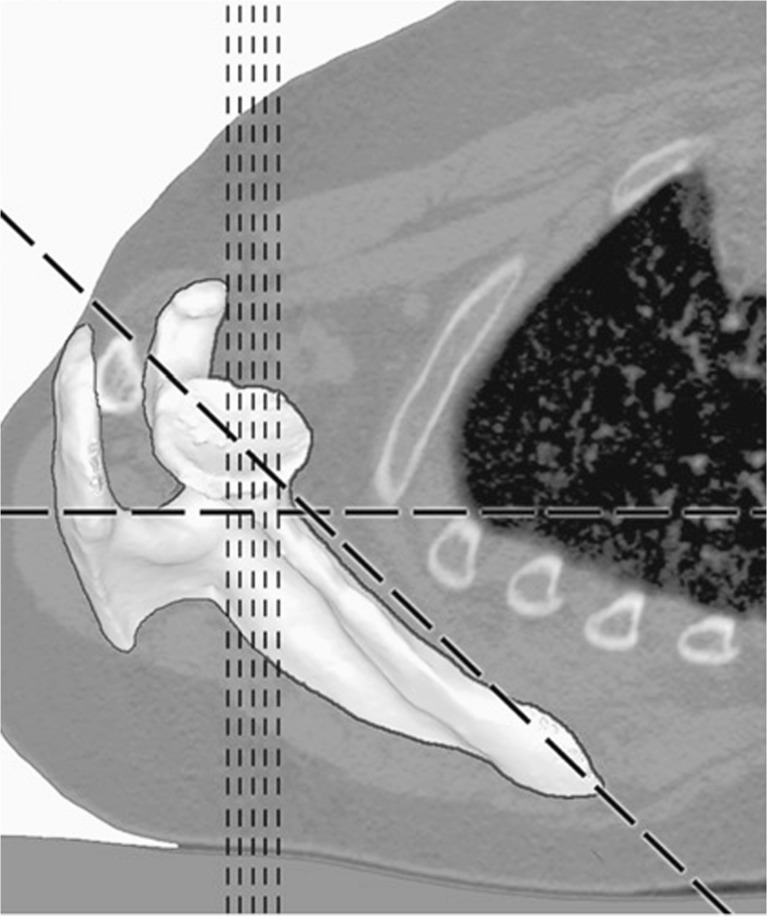
The difference between the orientation of standard axial 2-D CT scans (*vertical lines*) and the orientation of the scapula (*diagonal line*), the horizontal line indicates the orientation of the table the patient is laying on (*supine position*) [19]

Recently, it has become clear that the accepted gold standard for measuring glenoid version presents reliability issues [10, 19]. In healthy subjects, glenoid version measurements at different heights along the superior/inferior axis of the glenoid have been shown to differ amongst several investigators [20–23]. In general, glenoid version is measured at the mid glenoid level, which is less arbitrary when a CT scan of the shoulder has three or four cuts that traverse the glenoid [11]. Contemporary advancements in technologies allow for a larger selection of mid glenoid level slices. Furthermore, version measurements depend on slice orientation in creating 2-D images and variables that alter scapular orientation (Fig. 1). Especially in a child with an internal rotation contracture, anatomical variation of the glenohumeral joint and patient positioning in the gantry can greatly affect scapular orientation [10, 18, 19, 24]. These factors question the accuracy of these measurements. The findings of these measurements are important to the extent that surgeons use these numbers to indicate the need for surgical intervention. The purpose of this study was to evaluate how this known variability affects measurements of relevant parameters in a neonatal brachial plexus palsy population and evaluate the interobserver reliability of these measures. We hypothesized that glenoid version and percentage of the humeral head anterior to the scapular line as customarily measured in a clinical setting and most of the published literature would be altered by standardizing the measurements to the scapula’s orientation. We assessed our measurement outcomes by manipulating 3-D images to bridge the variance between clinical and anatomical version in a clinical setting.

## Materials and methods

With Institutional Review Board approval, 21 bilateral MRI scans (42 image sequences) of a consecutive series of children with internal rotation contractures were evaluated by four independent reviewers trained in a protocol, specific for slice selection and placement of reference axes for version measurements, between September 2011 and June 2012. Our study group existed of 7 boys and 14 girls, with a mean age of 3.8 years old (range: 1.3–12.3 years). The mean passive external rotation measured with the arm by the side was −13° (range: −70°–40°). The children included in this study were all candidates for surgical intervention, therefore MRI was conducted. The MRI studies were performed on a 1.5-T MRI unit (Signa; General Electric Medical Systems, Milwaukee, WI) with the use of Signa 5X software. Thin-section 3-D axial gradient-echo images (repetition time, 38 ms; echo time, 15 ms; flip angle, 20°; two signals acquired; 2-mm section thickness with 0-mm spacing, 120-mm field of view and 256 × 160 matrix) were made of both shoulders in the axial plane. Dedicated shoulder surface coils were used. Children were not moved in the gantry between securing images from the shoulder and corresponding elbow to preserve the coordinate system of the humerus. Children younger than 7 years of age are imaged under general anesthesia or sedation, according to our radiology and anesthesia department guidelines for children of that age. There were no complications as a result of the general anesthesia or sedation. Children were placed in the supine position with both arms placed against the side of the body and imaged simultaneously, parallel to each other and coaxial to the gantry as well as the contracture or body shape would allow. The total length of this protocol is 60–90 min.The MRI scans were anonymized and measured for glenoid version and percentage of the humeral head anterior to the scapular line by four independent reviewers. They included three musculoskeletal radiologists, one a fellowship-trained musculoskeletal radiologist with >10 years of experience outside of training (L.P.) and two senior residents in radiology fellowship training in musculoskeletal radiology (E.K.L.), and one fellowship-trained orthopaedic surgeon with 25 years of experience and a specialization in shoulder and elbow pathology (M.L.P.). The measurement protocol applied in this study is previously described by van de Bunt et al. [25]. Briefly, reviewers were trained to use our measurement protocol for 6 1-h sessions during a 6-week period before the study commenced. Reviewers were blinded to each other’s measurements and their own between clinical and reformatted measurements until the data collection was complete. Images were stored in DICOM (Digital Imaging and Communications in Medicine) format for further processing using radiologic software programs. General Electric Advantage Workstation with Version 4.4 reformatting software was used by the musculoskeletal radiologists and Osirix (Pixmeo, Geneva, Switzerland) was used by the orthopaedic surgeon. The first decision made in version measurement was choosing the mid glenoid slice [9, 11, 14, 26]. Reviewers were instructed to use the slice that represented the greatest glenoid girth, free from artifact and distortion. In this population, severe deformities and occasional artifacts can alter one’s choice of points from which to draw the line representing the anterior and posterior corners of the glenoid. The image slice selection chosen by the reviewer was recorded as part of the analysis.

Reference axes were chosen as conventionally described [11]. Retroversion was measured as the angle subtended by the glenoid line and a perpendicular to the scapular line on the posterior aspect of the scapula. Retroversion was assigned negative values and anteversion positive values as consistent with literature. Clinical version was measured on the axial images, as they were delivered from the technician through the picture archiving and communication system (PACS). Anatomical version measurements were made after the reformatting protocol according to the study by van de Bunt et al. [25]. The goal of reformatting is to realign reference axes so that the coronal, transverse and sagittal planes relate to the scapula and the glenoid. Reformatting alters the image slice sequence after which the orders are not directly comparable among reviewers so this was not recorded. The percentage of the humeral head anterior to the middle of the glenoid fossa (percentage of the humeral head anterior to the scapular line) was measured as described in earlier studies [7, 8, 18, 27].

### Data analysis

Means, standard deviations, and minimum and maximum values for numeric variables, as well as correlations between numeric variables, were computed for the involved and uninvolved sides separately, as well as for both sides together. The reliability of the quantitative measurements (glenoid version and humeral subluxation measured as percentage of the humeral head anterior to the scapular line) using the clinical and anatomical method was measured according to the method of Bland and Altman [28]. Pearson correlation coefficients were estimated between passive external rotation and glenoid version and percentage of the humeral head anterior to the scapular line measurements.

## Results

### Involved side

The mean clinical version from all reviewers on the involved side was −28.2° ± 16,1° (range: −53.5° to −6.3°). The maximum clinical retroversion measurement by a single reviewer was −60°, and the minimum version measurement was −2° (Table 1). In two instances, all four reviewers selected the same image slice to make their measurements. For four children, three out of four reviewers chose the same slice. For 14 children, 2 of 4 reviewers chose the same slice. For one subject, the reviewers all chose different slices. Using the stated image slice selection criteria, reviewers differed in their selection by a mean of 2.5 slices per child (range: 0–6). The percentage of the humeral head anterior to the scapular line averaged 26.9%, ranging from 0% to 57.8%.

**Table 1:** Clinically assessed glenoid version measurements by reviewers

	Age	External rotation	Clinical Version (°)						Anatomical version (°)					
			Reviewer				Mean	Range	Reviewer				Mean	Range
			1	2	3	4			1	2	3	4		
Mean	3.75	−13.33	−29.81	−28.86	−26.29	−28.00	−28.24	9.52	−25.38	−25.48	−23.71	−29.57	−26.04	11.95
Standard deviation	3.08	26.00	17.29	17.66	16.54	15.04	16.13	5.33	16.69	16.88	15.13	17.72	15.86	7.83
Minimum	1.13	−70.00	−54.00	−52.00	−60.00	−54.00	−53.50	2.00	−50.00	−56.00	−53.00	−66.00	−53.50	4.00
Maximum	12.31	40.00	−4.00	−2.00	−7.00	−7.00	−6.25	21.00	−2.00	−1.00	−4.00	−5.00	−4.00	38.00

The mean anatomical version from all reviewers was −26° ± 15.8° (range: −53.4° to −4.1°). The maximum anatomical retroversion measurement by a single reviewer was −66°, and the minimum version measurement was −1° (Table 1). The percentage of the humeral head anterior to the scapular line averaged 28.7%, ranging from 3.4% to 57.1%.

### Uninvolved side

Mean clinical version on the uninvolved side was −5.7° ± 3.1° (range: −11.2° to 1.1°), with maximum anteversion of 5° and retroversion of −15° as measured by a single reviewer. In one instance, all four reviewers selected the same image slice to make their measurements. For seven children, three out of four reviewers chose the same slice. For 12 children, 2 out of 4 reviewers chose the same slice. For one subject, the reviewers all chose different slices. Using the stated image slice selection criteria, reviewers differed in their selection by a mean of 2.4 slices per child (range: 0–10). The percentage of the humeral head anterior to the scapular line averaged 48.3%, ranging from 35% to 67%.

The mean anatomical version on the uninvolved side was −5.9° ± 2.8° (range: −12.5° to −1.7°). The maximum retroversion measurement by a single reviewer was −16°, and anteversion measurement was 1°. The percentage of the humeral head anterior to the scapular line averaged 47.4%, ranging from 41.5% to 54.8%.

### Measured difference between clinical and anatomical version measurement

**Fig. 2:**
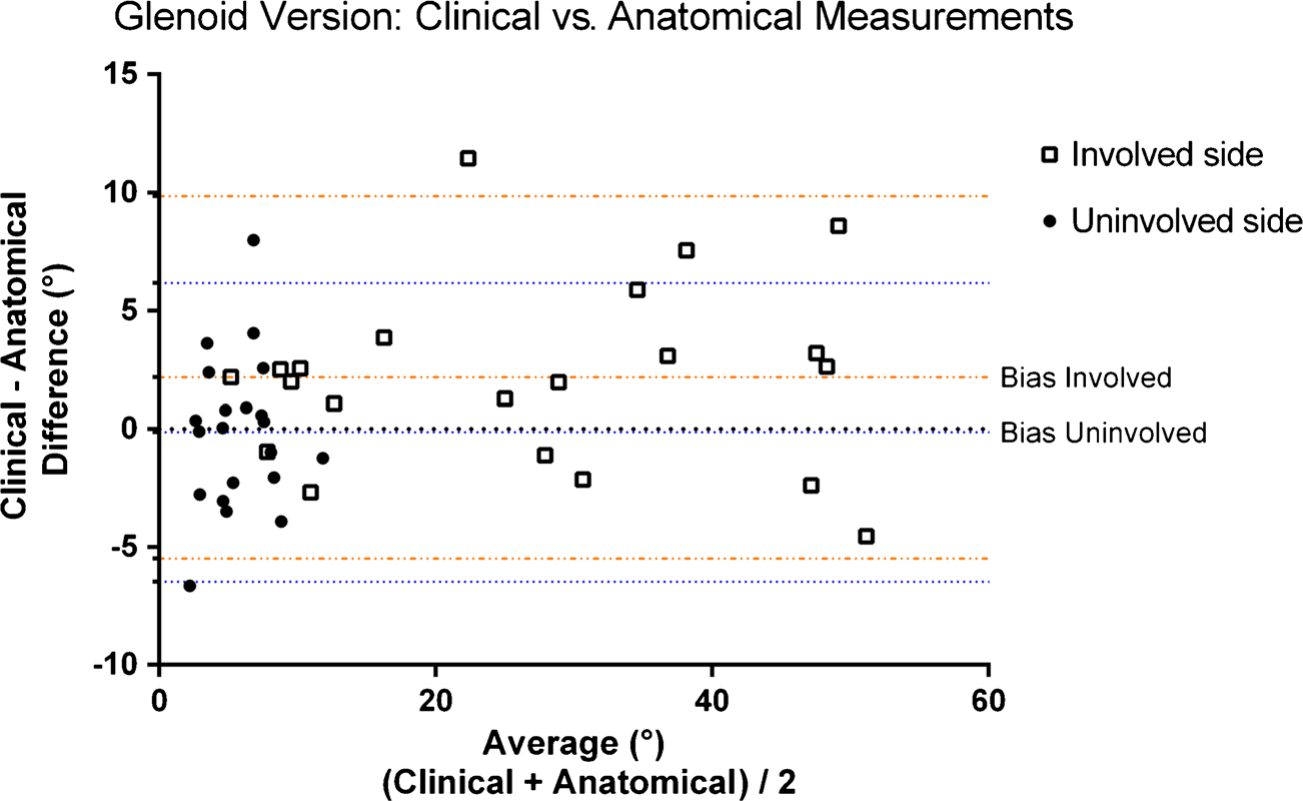
Bland-Altman plot demonstrates a bias of 2.19° (standard deviation [SD]: 3.92) over all measurements at the involved side and a bias of −0.14° (SD: 3.22) on the uninvolved side

The mean difference on the involved side between the clinical and anatomical versions across all children from all reviewers was 2.2° ± 3.9° (range: −4.5° to 11.5°) (Table 2). The range amongst the reviewers was −13° to 25°. On the uninvolved side, the mean difference between both measurement methods averaged −0.1° ± 3.2° (range: −6.7° to 8°). The range among the reviewers was −12° to 10°. The Bland-Altman plot demonstrated a bias (mean difference) of 2.19° (standard deviation [SD]: 3.92) over all measurements at the involved side and a bias of −0.14° (SD: 3.22) on the uninvolved side (Fig. 2). Nearly all measurements fell within the 95% level of agreement. Both methods demonstrated one outlier, which involved a different patient. The distribution of measurements is larger on the uninvolved side as opposed to the involved side.

**Table 2:** Glenoid version angle difference after 3-D reformatting

	Version angle difference: Involved side (°)						Version angle difference: Uninvolved side (°)					
	Reviewer				Mean	Range	Reviewer				Mean	Range
	1	2	3	4			1	2	3	4		
Mean	4.43	3.38	2.51	−1.57	2.19	14.03	1.00	−0.24	0.48	−1.81	−0.14	7.21
Standard deviation	6.96	8.09	5.53	6.89	3.92	7.66	3.54	4.56	3.79	5.50	3.23	3.81
Minimum	−8.00	−10.00	−10.60	−13.00	−4.55	6.00	−6.00	−10.00	−8.25	−12.00	−6.65	1.00
Maximum	25.00	25.00	12.84	11.00	11.46	37.00	10.00	9.00	7.00	6.00	8.00	13.40
Range	33.00	35.00	23.44	24.00	16.01	31.00	16.00	19.00	15.25	18.00	14.65	12.40

### Measured difference between clinical percentage of the humeral head anterior to the scapular line and percentage of the humeral head anterior to the scapular line measurement after reformatting

**Fig. 3:**
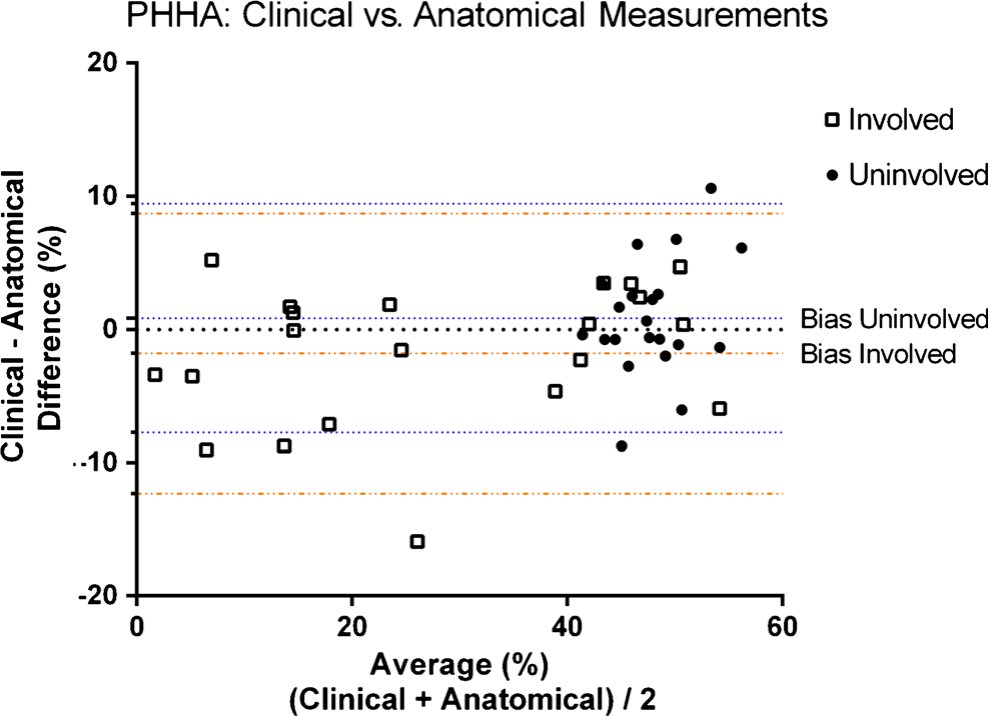
Bland-Altman plot demonstrates a bias of −1.77% (standard devation [SD]: 5.35) over all measurements at the involved side and a bias of 0.86% (SD: 4.38) on the uninvolved side. *PHHA* percentage of the humeral head anterior to the scapular line

The mean difference in percentage of the humeral head anterior to the scapular line after reformatting was −1.8% (range: -15.9% to 5.2%). The range amongst the reviewers was −30% to 23.1%. For the uninvolved side, the mean difference was 0.9% (range: −8.7% to 10.6%). The range amongst the reviewers was −12.6% to 23% (Table 3). The Bland-Altman plot demonstrated a bias (mean difference) of −1.77% (SD: 5.35) over all measurements at the involved side and a bias of 0.86% (SD: 4.38) on the uninvolved side (Fig. 3). Nearly all measurements fell within the 95% level of agreement, but both methods demonstrated one exception. The distribution of measurements is larger on the uninvolved side as opposed to the involved side.

**Table 3:** Percentage of the humeral head anterior to the scapular line (PHHA), difference after 3-D reformatting

	PHHA difference: Involved side (%)						PHHA difference: Uninvolved side (%)					
	Reviewer				Mean	Range	Reviewer				Mean	Range
	1	2	3	4			1	2	3	4		
Mean	−4.36	0.68	−3.22	−0.16	−1.77	15.52	−0.37	1.17	1.08	1.58	0.86	9.34
Standard deviation	6.39	9.96	4.76	11.07	5.35	6.76	5.40	6.32	4.58	7.62	4.38	5.69
Minimum	−17.18	−14.53	−13.61	−30.04	−15.91	6.71	−11.42	−9.60	−5.65	−12.62	−8.73	1.12
Maximum	4.31	21.83	4.91	23.16	5.19	34.12	10.37	16.70	12.52	22.95	10.62	26.60
Range	21.49	36.36	18.52	53.20	21.10	27.41	21.79	26.30	18.17	35.57	19.35	25.48

Passive external rotation measurements were correlated with anatomical glenoid version and percentage of the humeral head anterior to the scapular line measurements. For both anatomical version (*r* = 0.643; *P* = 0.002) and anatomical percentage of the humeral head anterior to the scapular line measurements there was a significant correlation (*r* = 0.611; *P* = 0.003). The passive external rotation measurements decreased, with an increase in glenoid retroversion and decrease of percentage of the humeral head anterior to the scapular line.

## Discussion

**Fig. 4:**
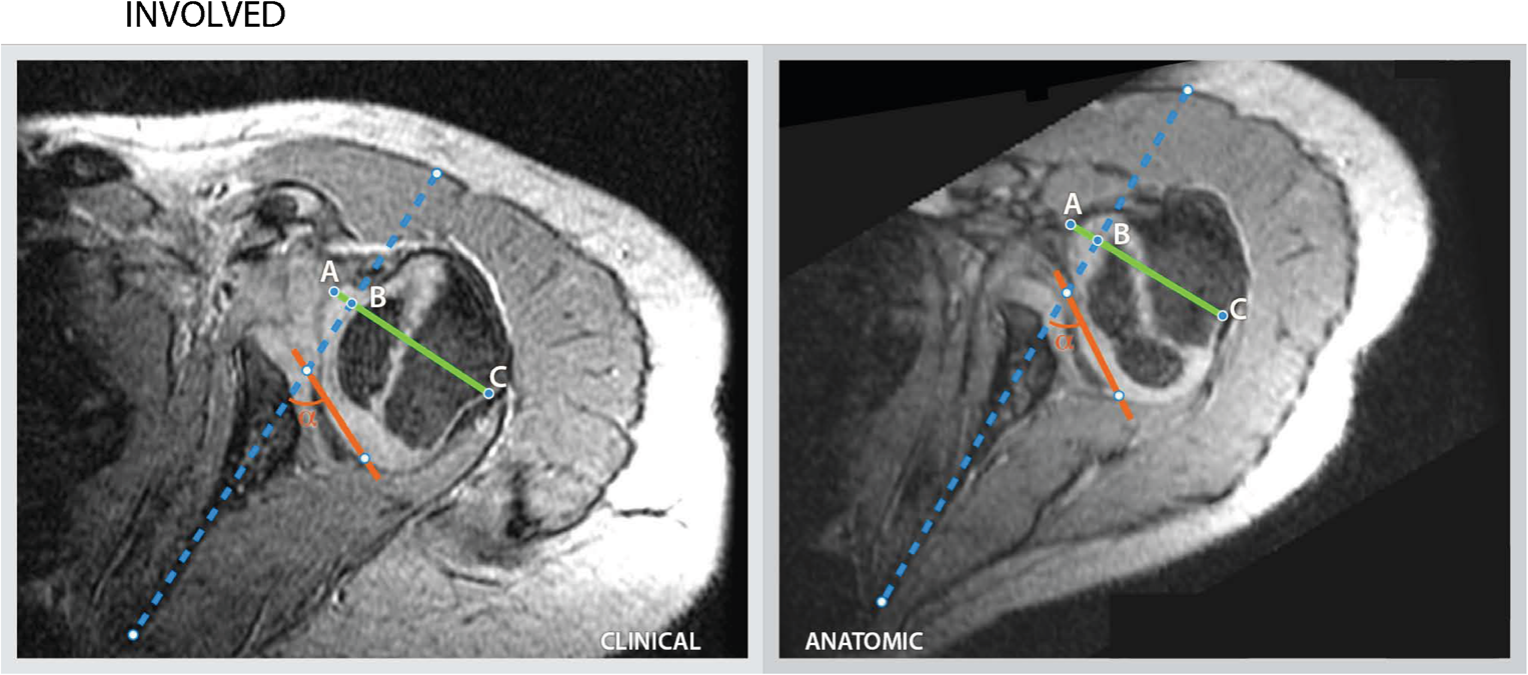
Measurements for child 1, performed by reviewer 3 for the involved with according measurement outcomes. The clinical and anatomical measurement outcomes for glenoid version (−90°) and percentage of the humeral head anterior to the scapular line (AB/AC * 100) are 21° and 11%, and 32° and 15%, respectively. *Dotted blue line:* scapular line, *Red line:* glenoid line, *Green line:* transverse line through the margins of the humeral head, perpendicular to the scapular line, *AB:* the distance of the humeral head, above the scapular line, *AC:* the distance of the green line perpendicular to the scapular line, *α:* The angle between the glenoid line and the scapular line (glenoid version = α - 90)

**Fig. 5:**
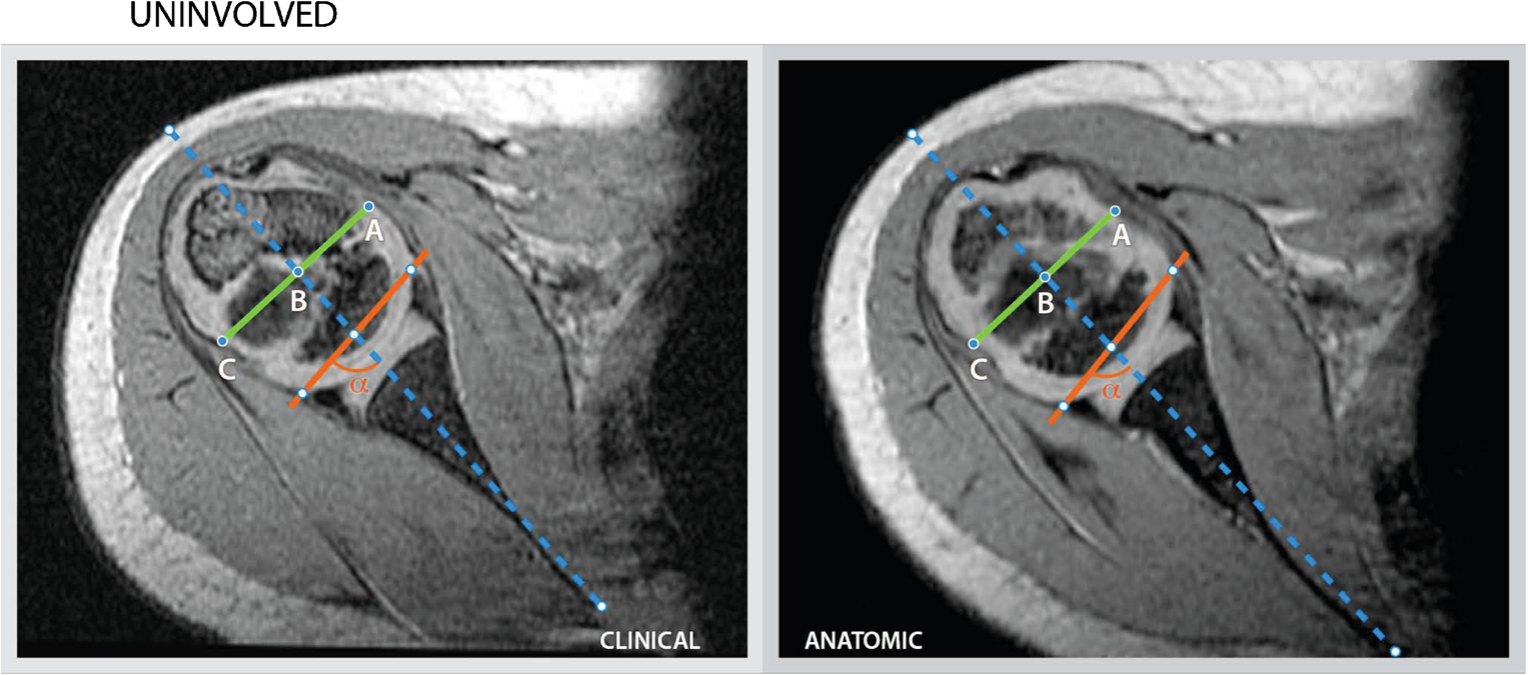
Measurement for child 1, performed by reviewer 3 for the uninvolved side with according measurement outcomes. The clinical and anatomical measurement outcomes for glenoid version (−90°) and percentage of the humeral head anterior to the scapular line (AB/AC * 100) are 7° and 49%, and 10° and 46%, respectively. *Dotted blue line:* scapular line, *Red line:* glenoid line, *Green line:* transverse line through the margins of the humeral head, perpendicular to the scapular line, *AB:* the distance of the humeral head (AB), above the scapular line, *AC:* the distance of the green line perpendicular to the scapular line, *α:* The angle between the glenoid line and the scapular line (glenoid version = α - 90)

The mean glenoid version and percentage of the humeral head anterior to the scapular line measurements observed for both the clinical and anatomical method are consistent with those in literature [2, 4, 7, 8, 18, 29, 30]. However, measurements can differ greatly for the same patient depending on technical factors of image acquisition and presentation in the clinical setting. The effect of 3-D reformatting on glenoid version measurements is best represented in the difference in measurement outcomes between both methodologies (Table 2, Figs. 4 and 5). In our study, we calculated a mean difference of 2.2°; however, the range in measured differences after 3-D reformatting within patients varied from −13° to 25° on the involved side as opposed to −12° to 10° on the uninvolved side. In no instance is the range in measured differences among observers lower than 7° on the involved side. The range in measured differences is >10° in 12 out of 21 measurements versus 5 out of 21 on the uninvolved side. These numbers represent overestimation as well as underestimation of the version angle using the clinical method, suggesting there is no systematic bias in the clinical version measurements. Over- or underestimation of the glenoid version angle can alter clinical decision-making regarding surgical indications. The 3-D reformatting protocol aims to introduce a systematic approach to improve the accuracy of measurement parameters relative to the scapula, in this study glenoid version and percentage of the humeral head anterior to the scapular line. Both the clinical and anatomical measurement methods were acceptably reliable in terms of inter- and intraobserver repeatability (precision); however, applying the reformatting protocol improves the accuracy by correcting for the variation in scapular orientation relative to the gantry due to patient positioning, variation in body habitus and posture. These concerns are especially relevant in this population with internal rotation contractures and other deformities of the shoulder. The 3-D reformatting protocol offers a clinically accessible recalibration tool for scapular orientation.

The most common decision in pursuing surgical treatment in this patient population is choosing between a humeral rotational osteotomy and a soft-tissue procedure. This decision is usually based primarily on the extent of glenohumeral deformity (inclusive of glenoid retroversion) and potential for remodeling [30–35]. The findings of this study suggest that these measurements can be misleading without correction for scapular orientation. Furthermore, minimizing measurement deviations allows for more reliable assessment of possible bony remodeling after surgical intervention. For percentage of the humeral head anterior to the scapular line measures, similar differences were found. The range in difference between clinical and anatomical measures varied in both directions (range: −30% to 23.1%). Similar clinical implications apply as to glenoid version measurements.

Various factors are involved in picking the best slice for version measurement. Assessing the mid-point slice, and how well the anterior/posterior edges are visualized, used to be less arbitrary when imaging techniques allowed for a limited number of slices. In the neonatal brachial plexus palsy population, severe osseous deformities can cloud mid-point assessment and appropriate border selection as illustrated in our study where slice selection proved to be inconsistent. Reviewers picked the same slice for version measurement in only 3 of the 42 possibilities. Note, there are fewer slices to pick as there would be in a skeletally mature scapula. These factors have yet not been taken into account when selecting the appropriate slice using contemporary imaging techniques. The observations made in our study regarding slice selection confirm this; however, it does not offer a consistent methodological solution.

With the software used for this study, the ability to manipulate 3-D models of the anatomy was limited. However, this software’s wide availability and open source make it a clinically useful tool for manipulating the 3-D anatomy of the scapula. For the coronal and transverse planes, the superior/inferior and anterior/posterior edges of the glenoid were readily identifiable and comparable with 3-D modeling. Some subjectivity is added in defining the sagittal plane neatly in line with the scapular body, since the scapular body is usually curved and rarely sits nicely in place. Scrolling back and forth through the 2-D images medial to the glenoid allows for approximation of the scapular body axis. This step admittedly would benefit from greater precision.

This study demonstrated comparable interobserver variability between the clinical and anatomical version measurements (Figs. 2 and 3).

For practical reasons, we did not investigate all separate components of the measurement process. Slice selection was recorded; however, variance in version from slice to slice was not assessed. Neither were reference points from which the measurement axes for scapular and glenoid lines are determined. Furthermore, intraobserver variability was not assessed, since we expected interobserver differences to exceed intraobserver differences. The focus of this study was on variability among observers related to the MRI scanning process and the clinical relevance accompanying the measured difference of the version angle after reformatting. The version values observed in this study are consistent with the literature, both for the involved and uninvolved side, as well as for clinical and anatomical methodologies. The precise value of version depends on the chosen definition of the scapular plane. When using 3-D models, which are so far solely available for study purposes, these measurements will become more precise [10, 19].

## Conclusion

The findings of this study show that glenoid version and percentage of the humeral head anterior to the scapular line are dependent on scapular orientation, as previously identified in other studies using scapular models in normal and arthritic shoulders, and extend this observation to the neonatal brachial plexus palsy population with internal rotation contractures. Measurements can differ greatly for the same patient depending on technical factors of image acquisition and presentation in the clinical setting. We recommend applying a 3-D reformatting protocol for accurate measurement of these parameters.
